# Perforin Is Detrimental to Controllinγ *C. muridarum* Replication In Vitro, but Not In Vivo

**DOI:** 10.1371/journal.pone.0063340

**Published:** 2013-05-14

**Authors:** Raymond M. Johnson, Micah S. Kerr, James E. Slaven

**Affiliations:** 1 Department of Medicine, Indiana University School of Medicine, Indianapolis, Indiana, United States of America; 2 Department of Biostatistics, Indiana University School of Medicine, Indianapolis, Indiana, United States of America; Midwestern University, United States of America

## Abstract

CD4 T cells are critical for clearing experimental *Chlamydia muridarum* genital tract infections. Two independent *in vitro* CD4 T cell mechanisms have been identified for terminating *Chlamydia* replication in epithelial cells. One mechanism, requiring IFN-γ and T cell-epithelial cell contact, terminates infection by triggering epithelial production of nitric oxide to chlamydiacidal levels; the second is dependent on T cell degranulation. We recently demonstrated that there are two independent *in vivo* clearance mechanisms singly sufficient for clearing genital tract infections within six weeks; one dependent on *iNOS*, the other on *Plac8*. Redundant genital tract clearance mechanisms bring into question negative results in single-gene knockout mice. Two groups have shown that perforin-knockout mice were not compromised in their ability to clear *C. muridarum* genital tract infections. Because cell lysis would be detrimental to epithelial nitric oxide production we hypothesized that perforin was not critical for *iNOS*-dependent clearance, but posited that perforin could play a role in *Plac8*-dependent clearance. We tested whether the *Plac8*-dependent clearance was perforin-dependent by pharmacologically inhibiting *iNOS* in perforin-knockout mice. *In vitro* we found that perforin was detrimental to *iNOS*-dependent CD4 T cell termination of *Chlamydia* replication in epithelial cells. *In vivo*, unexpectedly, clearance in perforin knockout mice was delayed to the end of week 7 regardless of *iNOS* status. The discordant *in vitro/in vivo* results suggest that the perforin’s contribution to bacterial clearance *in vivo* is not though enhancing CD4 T cell termination of *Chlamydia* replication in epithelial cells, but likely via a mechanism independent of T cell-epithelial cell interactions.

## Introduction

*Chlamydia trachomatis* genital tract infections have been, and continue to be, the most common bacterial sexually transmitted infections in Western societies [1]. Antibiotic-based public health measures have not decreased the incidence of infection and may be partially counterproductive, reducing *Chlamydia* immunopathology but also herd immunity [2]–[4]. Based on this epidemiologic reality many public health officials and sexually transmitted disease healthcare providers believe that a *Chlamydia* vaccine is the only intervention likely to reduce the incidence of infections and their associated monetary and personal costs.

Critical to a vaccine effort is defining the relevant host defense mechanism for protective immunity; *i.e.* the immunologic goal of vaccination. This is especially important for *Chlamydia* vaccines as early attempts with crude whole *Chlamydia* vaccines were associated with poor protection and excess immunopathology in humans and experimental primate models [5]. Investigations into *Chlamydia* genital tract protective immunity in the *C. muridarum* model have shown that CD4 T cells are critical to bacterial clearance [6], while CD8 T cells have been associated with immunopathology [7], [8].

Identification of an effector cell type does not equate a mechanism of protective immunity. The first identified mechanism for CD4 T cell-mediated termination of *Chlamydia* replication in epithelial cells was described in 1996. *In vitro Chlamydia*-specific Th1 cells terminated replication by inducing epithelial nitric oxide production to chlamydiacidal levels through a combination of IFN-γ and T cell-epithelial cell contact [9], [10]. However *in vivo* studies were discordant as mice deficient in inducible nitric oxide synthetase (*iNOS*) resolved *C. muridarum* genital tract infections [11], [12]; suggesting that nitric oxide was either irrelevant to bacterial clearance or redundant in the setting of a second independent clearance mechanism.

We recently showed that clearance of *C. muridarum* from the genital tracts of C57BL/6 mice during 8 weeks following infection was dependent on having either a functional *iNOS* or *Plac8* mechanism. Mice singly deficient nitric oxide production (treated with *iNOS* inhibitor N-monomethyl-L-arginine, MLA) or deficient in *Plac8* (genetic knockout) cleared *C. muridarum* genital tract infections at ∼6 weeks compared to ∼4 weeks for wild type mice. Dual deficient mice, genetically-deficient in *Plac8* and pharmacologically-deficient in *iNOS* activity (MLA treated) were effectively unable to clear a genital tract infection over 8 weeks with 11 of 12 mice still shedding an average of 1300 IFU/mouse on day 56. However, both *iNOS*-dependent (nitric oxide) and *Plac8*-dependent mechanisms were required for sterilizing immunity as viable *C. muridarum* could be recovered from genital tracts of *Plac8* knockout mice that had previously cleared a primary infection by delayed treatment with MLA [13]. This result mirrored that of Ramsey *et al* who showed that viable *C. muridarum* could be recovered from *iNOS*-knockout but not wild type mice that had previously cleared a genital tract infection when they were treated with the lymphocyte toxin cyclophosphamide [14].

Two previous studies have shown that perforin knockout mice were not compromised in their ability to clear *C. muridarum* genital tract infections. One study showed a trend toward less oviduct pathology in perforin knockout mice [15], the other a statistically significant reduction [8]. Because perforin knockout mice have an intact *iNOS*-clearance mechanism, it is possible that a role for perforin in *Plac8*-dependent clearance was masked by the *iNOS*-dependent clearance mechanism. With similar reasoning, we hypothesized that if perforin was necessary for the *Plac8*-dependent mechanism, then a perforin knockout mouse treated with MLA would not clear a *C. muridarum* infection over 8 weeks.

Unexpectedly we found that perforin knockout mice were compromised in their ability to clear *C. muridarum* genital tract infections, and that the role of perforin in clearance is not likely through enhancing CD4 T cell termination of *Chlamydia* replication by either of the two known *in vitro* mechanisms dependent on *iNOS* [10] and T cell degranulation [16].

## Materials and Methods

### Mice

4–5 week old female C57BL/6J and C57BL/6-Prf1^tm1Sdz/J^ mice were purchased from The Jackson Laboratories (Bar Harbor, MA). All mice were housed in Indiana University School of Medicine specific-pathogen-free facilities (SPF). All animal experiments were approved by the AAALAC-accredited Indiana University School of Medicine Animal Care and Use Committee performed in accordance with the United States Animal Welfare Act and the National Research Council’s Guide for the Care and Use of Laboratory Animals. Mice were lightly anesthetized with isoflurane for infections and vaginal swabs to minimize stress and discomfort.

### Genital Tract Infections

One week prior to infection mice were treated with 2.5 µg of medroxyprogesterone delivered subcutaneously (Depo-Provera, Pfizer Pharmaceuticals, New York, NY). Lightly anesthetized mice were infected vaginally with 5×10^4^ inclusion forming units (IFU) of *C. muridarum* biovar Nigg in 10 µl of SPG buffer. Mice were swabbed Tue-Thu to monitor the clearance of *C. muridarum* from the genital tract. Mice had access to water *ad libitum*, without or with 50 µM N-monomethyl-L-arginine (EMD4biosciences; Billerica, MA). Water was supplemented as needed and exchanged entirely for fresh solutions twice a week for the duration of the experiment as in the previous publication [13]. Power analysis based on our previous *Plac8* MLA publication [13] and a previous perforin knockout mouse study [15] showed that experimental groups of 5 mice had 97–99% likelihood to detect significant differences (*p value* <0.05) between groups if the assessment was done in the day 40–56 window post infection.

### Pathology Scoring

A simple scoring system was utilized to assess macroscopic genital tract pathology [13]. Hydrosalpinx was scored as 0, 1, or 2 reflecting no, unilateral, or bilateral hydrosalpinx respectively. Mice have a bi-fed uterus (2 uterine horns) that was similarly scored 0, 1, or 2 for hydro-uterus. The maximum pathology score for an individual mouse was 4.

### Media, T cell Expansion, Epithelial Cells, and Bacteria

T cell expansion cultures were grown in RPMI 1640 (Sigma) supplemented with 10% characterized fetal bovine serum (HyClone), 2 mM L-alanyl-L-glutamine (Glutamax I; Gibco/Invitrogen), 25 µg/ml gentamicin (Sigma), and 5×10^−5^M 2-mercaptoethanol (Sigma); referred to as RPMI CM. Immune splenocytes harvested from mice were plated at 12.5×10^6^ cells per well in tissue culture treated 12 well plates, in RPMI CM containing murine recombinant IL-1α (2 ηg/ml), IL-6 (2 ηg/ml), IL-7 (3 ηg/ml), IL-15 (4 ηg/ml), human recombinant IL-2 (100 units/ml), 20% 2° MLC, and 10 µg of UV-inactivated *C. muridarum* (∼2.5 IFU equivalents per splenocyte) as previously described [17]. Experiments were performed after the second passage/expansion. Polyclonal T cell populations were frozen at passages 3 and 4. C57epi.1 epithelial cells were cultured as previously described [17]. Mycoplasma-free *Chlamydia muridarum* (Nigg), previously known as *C. trachomatis* strain mouse pneumonitis (MoPn) (Nigg) was grown in McCoy cells as previously described [18].

### Cytokine ELISAs

3×10^6^ immune splenocytes were mock-activated (SPG buffer) or active with 7.5×10^6^ IFU UV-inactivated *C. muridarum* (1200J × 2 in a Spectrolinker XL-1000 UV crosslinker) in 48 well tissue culture plates in 0.7 ml of RPMI CM. Culture supernatant was harvested at 72 h and relative levels of IFN-γ, TNF-α, IL-13, and IL-10 in culture supernatants determined by ELISA using capture and biotinylated monoclonal antibody pairs with recombinant murine standards according to the manufacturer’s protocols. IFN-γ ELISA: XMG1.2 (Pierce-Endogen; Rockford, IL). TNF-α ELISA: TN3-19.12/C1150-14; IL-10 ELISA: JES5-2A5/SXC-1 (BD Biosciences; San Jose, CA). IL-13 ELISA: Ebio13a/Ebio1316H (Ebioscience; San Diego, CA). Detection was accomplished with Streptavidin-HRP (BD Biosciences) and TMB substrate (Sigma Chemical Co., St. Louis, MO).

### Flow Cytometry

Arbitrarily selected polyclonal T cell populations #5 (wild type) and #15 (perforin-deficient) were stained for 20 min on ice in RPMI CM with: PE-coupled 53-6.7 (CD8α) (BD Biosciences), FITC-coupled GK1.5 (CD4), FITC-coupled eBR2a (control antibody), PE-coupled LO-DNP-11 (control antibody) (Ebioscience; San Diego, CA). Cells were fixed with 1% paraformaldehyde after staining and analyzed on a FACSCalibur cytometer (BD Biosciences).

### Epithelial Cell Infections/*Chlamydia* Replication/Titration/Microscopy

C57epi.1 cell monolayers in 48 well plates were untreated or treated with IFN-γ (10 ηg/ml) for 10 h prior to infection. Wells were infected with 3 IFU per cell. After addition of *C. muridarum* the plates were spun at 300×g for 30 min. 4 h post infection the inoculums were removed and CD4 T cell clones were added in RPMI CM. 32 h post infection, wells were scraped, harvested with an equal volume of sucrose-phosphate-glutamate acid buffer (SPG buffer) and stored at −80°C until *C. muridarum* titers were determined on McCoy cell monolayers using an anti-*Chlamydia* LPS antibody (generous gift of Bobbi Van Der Pol and Jim Williams IUSOM) and FITC-labeled goat anti-mouse IgG (Rockland Immunochemicals; Gilbertsville, PA) as previously described [18]. For the DIC microscopy, arbitrarily selected polyclonal T cell populations, #5 (wild type) and #15 (perforin-deficient), frozen at the third passage were thawed/expanded then used under identical conditions as the *Chlamydia* replication/titration experiments except that instead of harvesting the monolayers at 32 h post infection the wells were fixed with 10% formalin and photographed using a Nikon Diaphot 200 inverted microscope equipped with a Diagnostic Instruments spot color camera using the plan20/0.4 DIC lens.

### Statistical Analysis

Summary figures for each experimental investigation are presented means and standard deviations (SD). Figure legends indicate the number of mice or independent T cell populations per data point in the experiment. Student’s two-tailed t-tests and Wilcoxon non-parametric tests, depending on data distribution, were used to assess significance of experimental data. Homogeneity of variances was assessed using a folded F-test. Statistical analysis of *Chlamydia* shedding was performed using repeated measures analysis of variance to test for differences between groups over time. All data were verified to meet analytic assumptions. Analyses were performed using SAS 9.3 (SAS Institute, Cary, NC).

## Results

### Perforin is not Likely Required for the Plac8-dependent Clearance Mechanism

We tested the role of perforin in *Plac8*-dependent clearance by treating perforin knockout mice with the *iNOS* inhibitor MLA and infecting them vaginally with *C. muridarum*. In our previous study *Plac8* knockout mice treated with MLA were effectively unable to clear a *C. muridarum* genital tract infection over 8 weeks, with 11 of 12 mice continuing to shed *C. muridarum* with an average intensity of 1300 IFU/swab; all wild type C57BL/6 mice treated with MLA, 11 of 11, cleared the infection by day 40. If the *Plac8*-dependent mechanism were perforin-dependent then perforin knockout mice treated with MLA would have a similarly compromised ability to clear *C. muridarum* from the genital tract.

**Figure 1:**
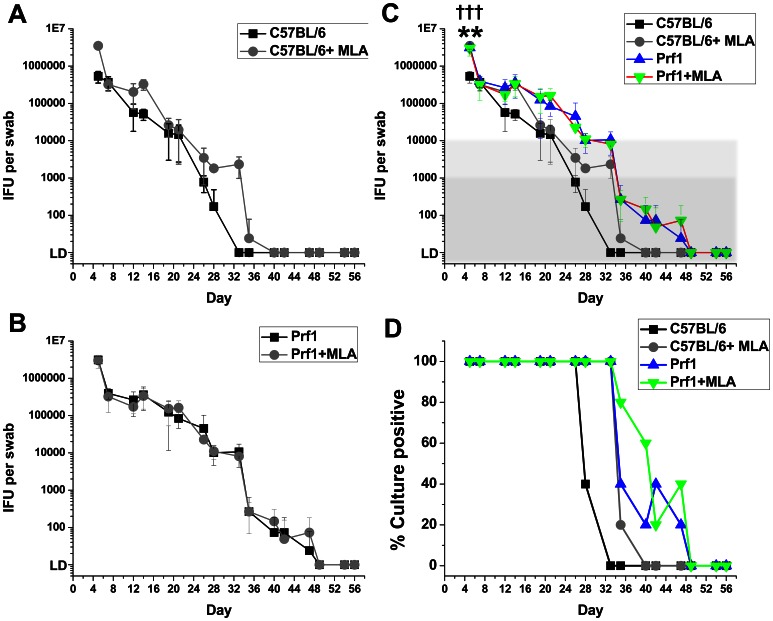
Pharmacologic inhibition of *iNOS* had no effect on clearance of *C.* muridarum from the genital tract of perforin knockout mice. Wild type C57BL/6J (10 mice; 5 untreated, 5 treated with MLA) and perforin knockout (10 mice; 5 untreated, 5 treated with MLA) female mice were treated with medroxyprogesterone, then infected vaginally one week later with 5×10^4^ IFU of *C. muridarum*. For MLA treatment groups the drinking water contained 50 µM MLA (*iNOS* inhibitor) one day before infection through day 56 post-infection. Genital tract shedding was monitored through day 56 post-infection. The 20 mice in the experiment were inoculated on the same day and monitored in parallel for the duration of the experiment. A) Shedding for C57BL/6J mice. B) Shedding for perforin knockout mice. C) Combined data. D) Combined data for % culture positive mice over 8 weeks. Differences in the clearance kinetics for C57BL/6J *versus* perforin knockout mice for the last 5 weeks of the experiment (last 4 logs of clearance, highlighted). Differences between the perforin knockout mice, without or with MLA treatment, and untreated wild type mice were statistically significant with an overall *p value* <0.0001. ** = *p value* <0.005 comparing wild type and perforin knockout mice at initial data point (day 5). ††† = *p value* <0.0005 comparing untreated wild type mice to wild type mice treated with MLA at initial data point (day 5).

C57BL/6J (H-2^b^) and perforin knockout mice (H-2^b^) were treated with medroxyprogesterone to synchronize estrous, then infected vaginally with 5×10^4^ IFU of *C. muridarum*, without or with addition of *iNOS* inhibitor MLA to the drinking water. Mice were swabbed twice weekly through 56 days, then assessed for genital tract pathology. Recovered IFUs from genital swabs were titered on McCoy monolayers (Fig. 1). Three of five wild type mice cleared the infection by day 28; the remaining two mice by day 33 (next sample collection). Addition of MLA to the drinking water of wild type mice lead to delay in clearance to day 40, the same day as our previous study. Perforin knockout mice cleared infections on Day 49, and neither the intensity of shedding nor time to clearance were affected by MLA treatment. Wild type and perforin knockout mice, under either treatment condition, were culture negative during the 8^th^ week of the experiment. Perforin knockout mice without or with MLA treatment had higher IFU shedding on initial measurement (day 5) and delayed clearance of *C. muridarum* from the genital tract compared to untreated wild type mice. At the end of experiment all mice were sacrificed and histopathology was scored. As seen in our previous study, treating wild type C57BL/6 mice with MLA did not change the severity of reproductive tract pathology (*data not shown*). Perforin knockout mice trended toward less pathology than wild type mice (50% less; *p value* = 0.17) consistent with previously published data [8], [15].

### Delayed Clearance in the Perforin Knockout Mice is not Due to T cell Polarization

**Figure 2:**
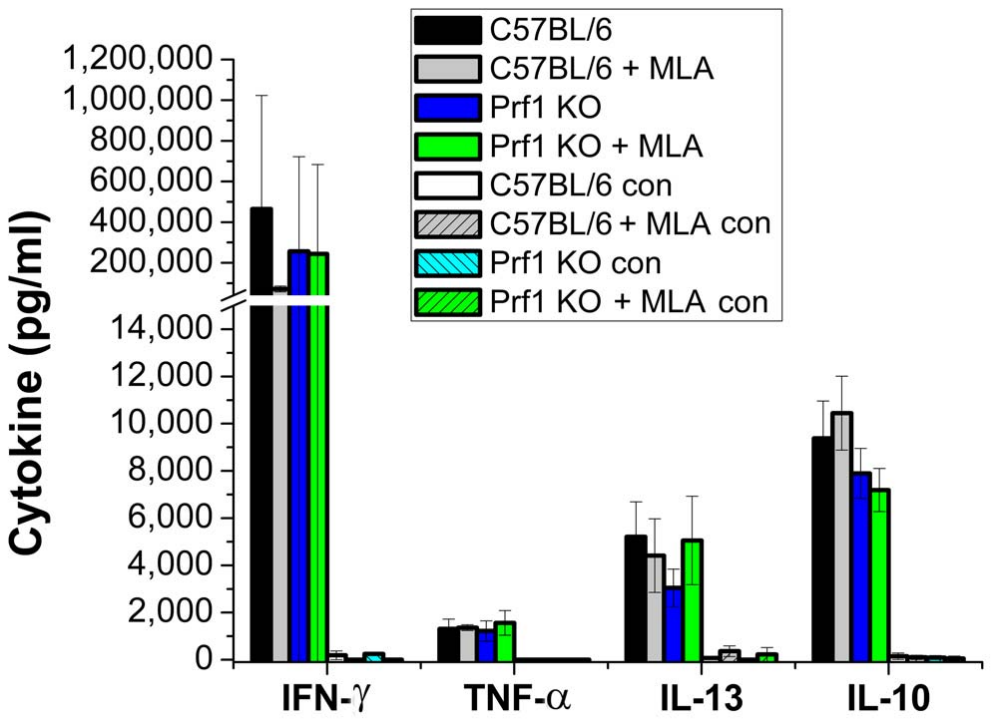
Cytokine profile of immune splenocytes from each experimental group infigure 1 (20 independent splenocyte populations) activated with UV-inactivated *C. muridarum.* 3×10^6^ splenocytes were activated with 7.5×10^6^ UV-inactivated *C. muridarum* (multiplicity of 2.5 IFU per splenocyte) or mock-activated with equivalent volume of SPG buffer (con = control) in triplicate wells. Supernatants were collected at 72 h and analyzed by ELISA. Data representing each mouse strain/treatment condition were aggregated and compared to the aggregated data for the untreated C57BL/6J mice. Comparisons were made for each cytokine. There were no statistically significant differences in cytokine polarization between wild type and perforin knockout mice with or without MLA treatment.

Spleens were harvested from all 20 mice on Day 56 to determine whether there was any difference in cytokine polarization related to the absence of perforin or treatment with MLA. Splenocytes were mock-activated (SPG buffer) and activated *in vitro* with UV-inactivated *C. muridarum* (stock in SPG buffer); culture supernatants collected at 72 h for cytokine analysis (Fig. 2). Protective immunity in experimental *Chlamydia* vaccine studies has previously been shown to been dependent on Th1 T cells [19] making IFN-γ and TNF-α [20]. IL-10 SNPs have been associated with susceptibility to *Chlamydia*-mediated tubal infertility in humans [21]. The Th2 cytokine IL-13 has been found to be detrimental in *C. muridarum* pulmonary infections [22]. There was no significant difference in IFN-γ, TNF-α, IL-10, or IL-13 between any of the mice or treatment conditions. MLA had no effect on cytokine polarization. Cytokine polarization does not explain the delayed clearance in perforin knockout mice.

### Perforin is Detrimental to the iNOS-dependent Clearance Mechanism *in vitro*

**Figure 3:**
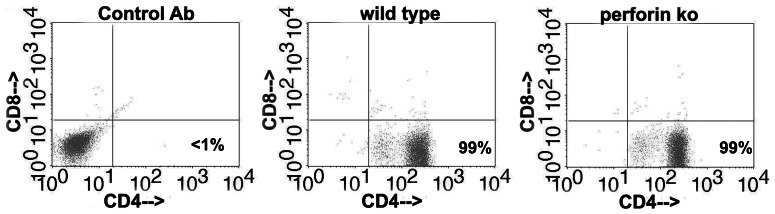
*Chlamydia*-specific polyclonal T cell populations derived from immune mice are 99% CD4^+^CD8^−^ after three passages with UV-inactivated *C. muridarum* and supporting cytokines regardless of perforin status.

**Figure 4:**
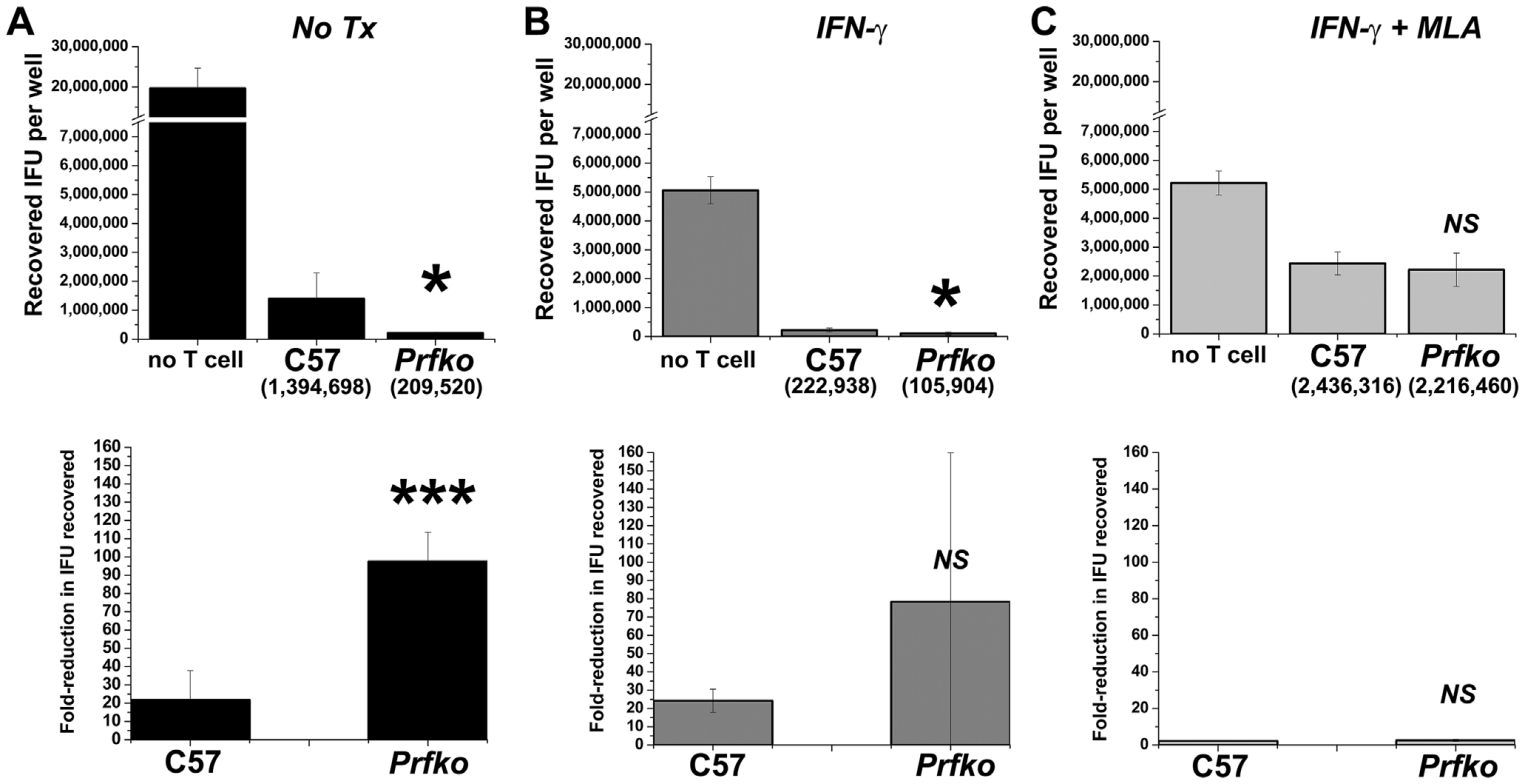
Perforin-deficient CD4 T cells are superior to wild type CD4 T cells for terminating *C.* muridarum replication in epithelial cells. Top panels show IFU recovered per well, with means for the experimental wells shown in parentheses below their respective bars. The bottom panels show the T cell-mediated fold-reduction in IFU (calculated as IFU recovered from the “no T cell” control wells/IFU recovered in the experimental wells). A) Untreated C57epi.1 monolayers. B) C57epi.1 monolayers treated with 10 ηg recombinant murine IFN-γ for 10 h prior to infection. C) C57epi.1 monolayers treated with 10 ηg recobinant murine IFN-γ and 1 mM MLA for 10 h prior to infection. All monolayers were infected with *C. muridarum* at 3 IFU per cell. Inocula were removed 4 h later and infected epithelial cells were co-cultured without (no T cells) and with 2.5×10^5^ T cells in the absence and presence of MLA as indictated. 32 h post infection supernatants were collected and the wells harvested; *C. muridarum* was quantified on McCoy monolayers. In a single experiment, each experimental condition (no treatment, IFN-γ pretreatment, IFN-γ-MLA pretreatment) was done as triplicates for each of the 10 T cell lines and the “no T cell” control. Comparisons were made between aggregated data for the 5 wild type T cell lines (C57) and the 5 perforin knockout T cell lines (PrfKo) for each experimental condition. * = *p value* <0.05; *** = *p value* <0.0005; *NS* = not statistically significant.

**Figure 5:**
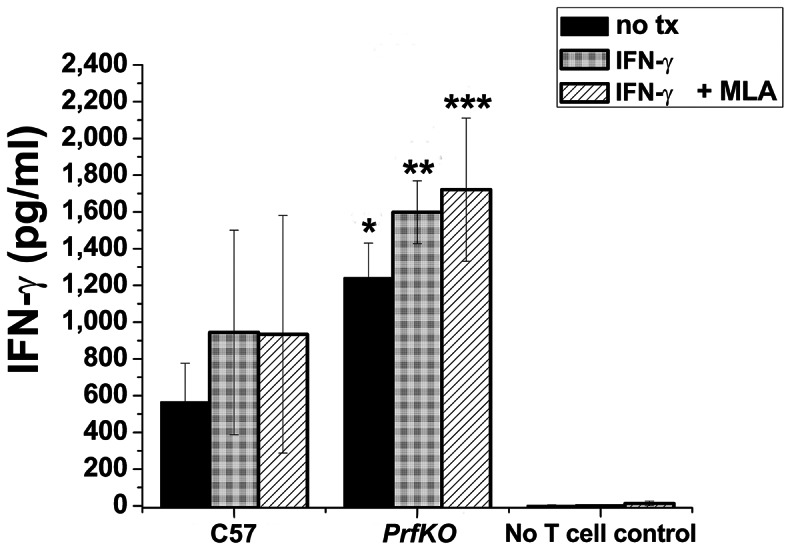
IFN-γ levels for each treatment condition in fiγure 4 as determined by ELISA. MLA treatment had no statistically significant effect on wild type or perforin-deficient T cells compared to the untreated control for each T cell type. * are for comparisons to ‘C57 no tx’ for all conditions; * = *p value* <0.05; ** = *p value* <0.005; *** = *p value* <0.0005; *NS* = not statistically significant.

**Figure 6:**
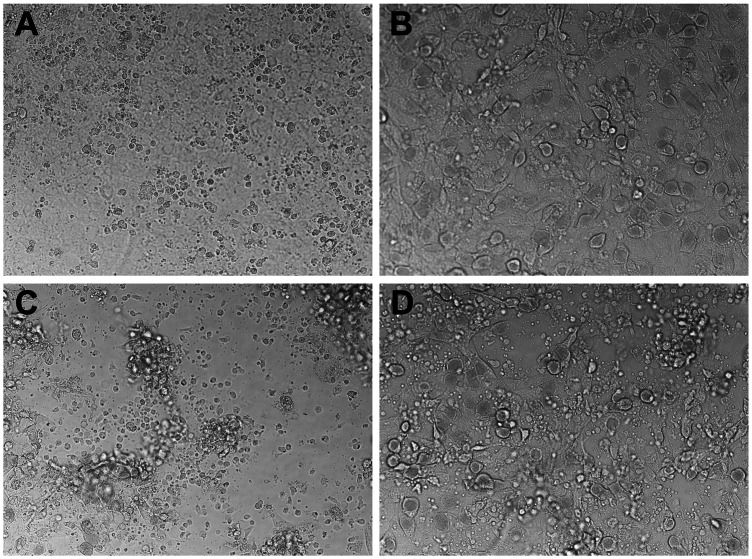
Perforin-deficient CD4 T cells have a lesser cytopathic effect on infected C57 epithelial monolayers. A) Uninfected C57epi.1 monolayer at 32 h post mock infection (overgrown confluent monolayer). B) C57epi.1 monolayer 32 h post infection with *C. muridarum* at 3 IFU per cell. C) Infected C57epi.1 monolayer co-cultured with wild type polyclonal CD4 T cells at an effector to target ration of 1.25∶1 at 32 h post infection. D) Infected C57epi.1 monolayer co-cultured with perforin-deficient polyclonal CD4 T cells at an effector to target ration of 1.25∶1 at 32 h post infection. Representative DIC images at 200x magnification.

Immune T cells from the 5 untreated wild type and 5 untreated perforin knockout mice in the experiment shown in figure 1, harvested on day 56, were separately expanded *in vitro* for 2 passages using UV-inactivated *C. muridarum* and supporting cytokines (*see materials and methods*). The resulting *Chlamydia*-specific polyclonal T cell populations were 99% CD4 T cells (Fig. 3). We tested the ability of these polyclonal populations to terminate *C. muridarum* replication in oviduct epithelial cell line C57epi.1, untreated or pretreated with IFN-γ, in the absence or presence of the *iNOS* inhibitor MLA. Epithelial cells were infected with 3 IFU per cell; 4 h later polyclonal T cells were added to the wells at an effector to target ratio of 1.25 to 1 (250 k T cells to ∼200 K epithelial cells). 32 h post infection supernatants were collected, and the wells harvested by scraping in SPG buffer to determine the extent of *C. muridarum* replication; IFU recovered were quantified on McCoy monolayers (Fig. 4). The amount of IFN-γ produced by T cells under each experimental condition was determined by ELISA (Fig. 5). Though not quantifiable, examination of the wells under phase contrast microscopy showed an obvious relative preservation of the epithelial cell monolayer at 32 h post infection in the wells containing perforin-deficient T cells compared to wild type T cell wells (Fig. 6), consistent with absence of the perforin the principal effector molecule for T cell-mediated cytolysis. Because there were more visible inclusions but fewer recovered IFU from the wells containing perforin-knockout T cells, it is reasonable to suppose that many EB within those visible inclusions were dead. As seen in our previous study [16], overnight treatment of the C57epi.1 epithelial monolayer prior to infection reduced replication by ∼4-fold. In untreated monolayers, perforin-deficient CD4 T cells were 5 times more efficient at terminating *C. muridarum* replication in epithelial cells than were wild type T cells. That difference in efficiency was blunted somewhat by pretreating the epithelial cell monolayers with IFN-γ, boosting epithelial nitric oxide production and MHC class II expression [17]. Both wild type and perforin-deficient early passage polyclonal T cell populations used the *iNOS*-dependent mechanism to terminate replication as addition of MLA to the experimental wells essentially abrogated their ability to terminate *Chlamydia* replication. In the presence of 1 mM MLA both polyclonal T cell populations reduced recovered IFU by only 2-fold. This result suggests that CD4 T cells capable of *iNOS*-independent termination of replication were present in numbers below that necessary to efficiently control replication in the presence of MLA. Consistent with, or in addition to, their greater efficiency in terminating replication, perforin-deficient T cells made ∼2-fold more IFN-γ under all experimental conditions (see Fig. 5).

## Discussion

Studies of protective immunity against *Chlamydia* infections of the genital tract have been confounded by the existence of three independent and singly sufficient mechanisms for clearing *C. muridarum* (reviewed in [23]). The first mechanism discovered is dependent on T cell- epithelial cell contact and IFN-γ−mediated up regulation of epithelial nitric oxide production to chlamydiacidal levels [10]. The second mechanism described is T cell-independent once established [24]; it is dependent on *Chlamydia*-specific antibodies [25] and likely Fc receptors [26]. The third mechanism discovered is dependent on *Plac8* [13] and likely CD4 T cell degranulation [16].

Based on the previous work of others showing no difference in clearance between C57BL/6 and perforin knockout mice [8], [15], and our work showing a dramatically diminished ability to clear *C. muridarum* in *Plac8* knockout mice treated with MLA [13], we sought to definitively test whether the *Plac8*-dependent mechanism was dependent on perforin by comparing clearance in perforin knockout mice without and with *iNOS* inhibitor treatment. Our experiments show that perforin knockout mice had delayed clearance compared to wild type mice, and that MLA treatment had no effect on clearance in the perforin knockout mice. Delayed clearance in perforin knockout mice could not be attributed to a difference in cytokine polarization and was not associated with greater immunopathology.

The finding of delayed clearance in perforin knockout mice differs between Perry *et al* [15] and our current study, likely reflecting the inoculating dose; 1500 IFU per mouse and 50,000 IFU per mouse respectively. In support of that possibility, Perry *et al* saw no difference in wild type and perforin knockout mice genital tract IFU at their initial time point; in our study there was 6-fold higher IFU in the perforin knockout mice than wild type mice on day 5. Our results also differ from those of *Murthy et al*. [8] who used an inoculating dose of 50,000 IFU, but treated mice with medroxyprogesterone on day T-10 and day T-3 prior to infection. In their model, IFU recovered on day five are ∼20,000/swab while our recovered IFU on day 5 are ∼2,000,000/swab. It is likely that the higher effective inoculating dose in our experimental model (based on inoculum size or hormonal manipulation) revealed defects in clearance not seen in the two other models.

**Figure 7:**
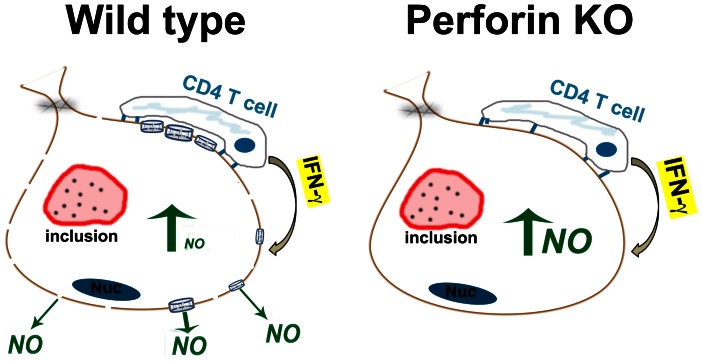
The toxic bag paradigm for CD4 T cell termination of replication in epithelial cells. *Chlamydia-specific* wild type T cells damage epithelial membrane integrity by inserting perforin channels and activating apoptotic pathways within target cells. Effectiveness of nitric oxide termination of replication is concentration dependent. Perforin-mediated damage to the membrane likely lowers the effective concentration of nitric oxide within the target cell, diminishing the relative potency of wild type CD4 T cells compared to perforin-deficient CD4 T cells. **Nuc** = nucleus.

The data in figure 4 show that perforin is detrimental to the *iNO*S-dependent mechanism for terminating *Chlamydia* replication in epithelial cells, either because cytolysis of the epithelial target decreases T cell activation and IFN-γ production, or more likely, inserting perforin channels (punching holes) in the epithelial membrane lowers the effective concentration or duration of exposure of *Chlamydia* to epithelial cell-produced intracellular nitric oxide. 5-fold more efficient replication termination by perforin-deficient T cells occurred in the setting of diminished cytolysis (see Fig. 6). More intact epithelial cells with visible inclusions were seen in perforin-deficient T cell wells, but fewer viable IFU were recovered from them suggesting that EB within those inclusions were dead; a counter intuitive result for conventional intracellular pathogen host defense paradigms. Because the IFN-γ levels were low (1–2 ηg/ml), and only increased ∼2-fold in perforin-deficient experimental wells, we postulate that the 5-fold greater potency of perforin-deficient T cells reflects a detrimental effect of perforin on functional levels of microbicide within infected cells. In support of that postulate, we have previously shown that addition of IFN-γ to our reproductive tract epithelial cell lines 4 or more hours after infection had no effect on *C. muridarum* replication or on the replication of the much more IFN-γ sensitive human *C. trachomatis* serovars D and L2 [27]. In these current experiments we added the T cells to the infected monolayers 4 h post-infection. We have also previously shown in 4 of 4 *Chlamydia*-specific T cell clones that the earliest recognition of untreated infected epithelial cells as measured by IFN-γ production is between 15–18 h post-infection [17], [28]. In our current experiment, IFN-γ in meaningful levels were likely non-existent until ∼20 h into the experiment, a time point at which the large majority of *C. muridarum* replication is already complete in C57epi.1 cells [17]. As a working model we propose that CD4 T cell-mediated termination of *Chlamydia*-replication in epithelial cells occurs via a toxic bag type mechanism. T cells form conjugates with infected epithelial cells, activating targeted cells to make nitric oxide; punching holes in the plasma membrane and/or lysing the epithelial cell is detrimental to this mechanism (Fig. 7).

Perforin-deficient mice treated with MLA did not behave like *Plac8*-deficient mice treated with MLA. Our previous experience was that *Plac8* knockout mice treated with MLA could not clear *C. muridarum* within 8 weeks, with substantial levels (1300 IFU/swab) of ongoing IFU shedding at the end of week 8, day 56. All perforin-deficient mice, untreated or treated with MLA, cleared their infection by day 49 with no measurable IFU shedding during the 8^th^ week of the experiment. Because untreated perforin knockout mice unexpectedly differed from C57BL/6 mice, we could not performed the planned statistical analysis comparing perforin-deficient mice to perforin-deficient mice treated with MLA to assess whether perforin was critical to the *Plac8*-dependent mechanism. However, it is clear from the data here that there was no difference in clearance between a perforin knockout mouse and a perforin knockout mouse treated with MLA. Had the *Plac8*-dependent mechanism been dependent on perforin, we would have expected MLA-treated perforin knockout mice to shed *Chlamydia* throughout the 8^th^ week of the experiment; they did not. The delayed clearance in untreated or MLA-treated perforin knockout mice compared to wild type mice is unlikely to represent a compromised *Plac8*-dependent clearance mechanism that is strongly associated with the *in vitro* degranulation-dependent termination mechanism [13], [16]. Perforin’s contribution to bacterial clearance is not likely occurring through enhancing CD4 T cell termination of *Chlamydia* replication in epithelial cells as it does not appear to relevant to the *Plac8*-dependent mechanism and is detrimental to the *iNOS*-dependent mechanism.

T cells, mast cells, eosinophils, basophils, CD4negative NKT cells and NK cells express perforin. NK cells recognize and lyse infected epithelial cells [29], [30], but NK cells by themselves have only a modest ability to terminate replication in epithelial cells *in vitro* (∼2-fold) [30]. However, cytolysis of infected cells by any means would expose intracellular *Chlamydia* EB to extracellular defense mechanisms including complement. *Chlamydia trachomatis* EB activate the alternative complement pathway and C3b covalently binds to MOMP [31], and early components of complement upstream of the membrane attack complex neutralize *Chlamydia* EB *in vitro* [32]. While existing data argue against complement being singly sufficient for clearing genital tract infections, as T cell deficient mice sufficient in complement cannot clear infections, complement could logically contribute to substantial reductions in viable extracellular EB in genital tract secretions. A recent study in a murine *C. psittaci* model showing a major contribution of C3 to protective immunity [33] raises the question of whether previous unpublished negative data based on cobra venom depletion of the complement membrane attack complex referred to in Williams *et al* [34] sufficiently addressed the role of early complement components in innate *Chlamydia* immunity.

### Conclusion

During experimental *C. muridarum* genital tract infections perforin plays a complex role. Others have shown that it contributes to the immunopathogenesis of scarring [8]. Here we show that perforin contributes to bacterial clearance, likely by a mechanism that does not involve CD4 T cell interactions with infected epithelial cells.
