# Correction: Perforin Is Detrimental to Controlling *C. muridarum* Replication In Vitro, but Not In Vivo

**DOI:** 10.1371/annotation/b7213da3-498c-43bf-b42c-1f22934e17dd

**Published:** 2013-07-02

**Authors:** Raymond M. Johnson, Micah S. Kerr, James E. Slaven

The word "Controlling" in the title was misspelled. The correct title is: Perforin Is Detrimental to Controlling *C. muridarum* Replication In Vitro, but Not In Vivo. 

The correct citation is: Johnson RM, Kerr MS, Slaven JE (2013) Perforin Is Detrimental to Controlling *C. muridarum* Replication In Vitro, but Not In Vivo. PLoS ONE 8(5): e63340. doi:10.1371/journal.pone.0063340. 

